# Large Electromechanical Response and Field‐Induced Shape Memory Effect in Ferroelectric Ceramics

**DOI:** 10.1002/advs.202410580

**Published:** 2025-01-29

**Authors:** Menglu Li, Weili Li, Wenping Cao, Nuo Xu, Wenqi Li, Weidong Fei

**Affiliations:** ^1^ School of Materials Science and Engineering Harbin Institute of Technology Harbin 150001 P. R. China; ^2^ School of Light Industry Harbin University of Commerce Harbin 150028 P. R. China

**Keywords:** bending deformation, field‐induced strain, Na_0.5_Bi_0.5_TiO_3_, PbTiO_3_, shape memory effect

## Abstract

Electric‐field‐induced shape memory effect has potential applications in electromechanical actuator. Here, this study proposes the a phase structure design routine in (1‐*x*)(75Na_0.5_Bi_0.5_TiO_3_‐25SrTiO_3_)‐*x*PbTiO_3_ ceramics to obtain large electromechanical response and shape memory effect. It is found that the shape memory effect is closely related to the bending deformation induced by asymmetric polarization between positive and negative electrodes, which is resulted from the reductions of Bi^3+^ and Pb^2+^ because of electron injection from negative electrode. In addition, the content of ferroelectric phase with P4 mm structure and *c/a* ratio increase with PbTiO_3_ content increasing, and the coercive field of the ceramics is also enhanced by the PbTiO_3_ addition, Combing the crystal structure and chemical composition, the field‐induced deformation achieves 2.77% and shape memory effect is 1.75% at 80kV cm^−1^ and 0.1 Hz in the ceramic with the content of PbTiO_3_ is 40%.

## Graphical abstract

A large asymmetry field‐induced deformation (2.77%) and shape memory effect (1.75%) have been found in (1‐x)(75Na_0.5_Bi_0.5_TiO_3_‐25SrTiO_3_)‐xPbTiO_3_. Because of the electron injection, the two sides of ceramics are in different polarization state and generate a bending deformation over the ceramic.

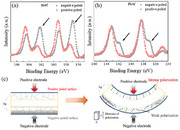

## 1 Introduction

Electric‐field‐induced strain (EFIS) effect of ferroelectric ceramics has been intensively studied because their important applications in actuators,^[^ ^1^, ^2^, ^3^ ^]^ sensors^[^ ^4^, ^5^, ^6^ ^]^ and energy storage.^[^ ^7^, ^8^, ^9^ ^]^ The EFIS effect of ferroelectric materials mainly originates from intrinsic part and extrinsic part. The intrinsic EFIS includes electrostrictive and piezoelectric effects, while the extrinsic part is closely related to domain switch and. In addition, some perovskite ferroelectric will undergo phase transition between antiferroelectric and ferroelectric, which will induce the volume change.^[^ ^10^, ^11^ ^]^

In addition, some ferroelectric ceramics exhibit strain shape memory effect that can be maintained a certain amount of strain after the external electric field is removed. When the reverse electric field is applied, the memorized strain can be restored.^[^ ^12^ ^]^ One strategy to design the strain memory effect in piezo ceramics is the phase transition from antiferroelectric to ferroelectric (FE), such as PZT‐based single crystal.^[^ ^12^, ^13^, ^14^ ^]^What's more, the tristate ferroelectric is also one of the solutions to achieve the strain memory. Zhang et al.^[^ ^15^ ^]^ have found that the relaxor‐ferroelectric state in Fe‐ Nb co‐doped Bi_0.5_Na_0.5_TiO_3_ (BNT) ceramics was thermodynamically intermedia state between the two FE remanent states and results in a memory effect. In addition, the defective ferroelectric ceramics potentially exhibit strain memory effect. Lv et al.^[^ ^16^ ^]^ reported a strain memory of 0.24% by introducing and aligning the defective poles in BiFeO_3_‐based ceramics. The combination of atom‐scale defective engineering and meso‐scale domain engineering achieved an ultrahigh electrostrain of 2.3% at 220 °C and a strain memory of 1.1% at 25 °C.^[^ ^17^ ^]^ Recently, He et al.^[^ ^18^ ^]^ have demonstrated that a bending strain introduced by external electric field in textured bismuth tungstate ceramics is remained when the external electric filed is removed, which results in a strain shape memory of 0.77%. And the strain memory effect is affected by the thickness of ceramics.^[^ ^19^ ^]^ However, the detail mechanism of the strain memory effect, such as the roles of the phase structure and chemical composition, is still needed to be studied.

Bi_0.5_Na_0.5_TiO_3_‐SrTiO_3_ (BNT‐ST) is a series of ceramics which exhibits relaxor‐ferroelectric state at room temperature, and morphotropic phase boundary (MPB) exits as the content of SrTiO_3_ of ≈25% and the phase transition from RE P4bm to R $\bar{3}$ C results in a large strain in 75BNT‐25ST ceramics.^[^ ^20^, ^21^ ^]^ In the present study, PbTiO_3_ with the symmetry of P4 mm is induced to the 75BNT‐25ST ceramics to study the influence of phase structure on the field‐induced shape memory effect of the ceramics. A large electric‐field‐induced shape memory effect is obtained in the ceramic plates, and a mechanism of asymmetry polarization is put forward. It is believed that the results presented in this paper are useful for designing electric‐field‐induced shape memory effects based on phase structure and chemical composition in ferroelectric ceramics.

## 2 Results and Discussion

### 2.1 Phase Structure and Domain Morphology

**Figure 1:**
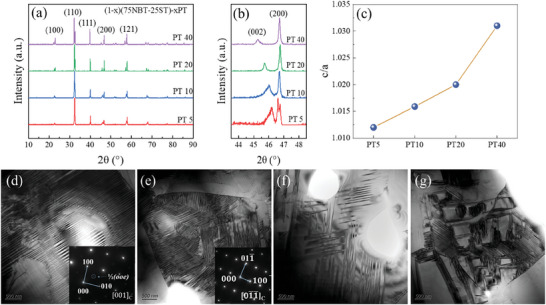
Phase structure and domain morphology of (1‐*x*)BNST‐xPT ceramics. a) XRD patterns of (1‐*x*)BNST‐xPT ceramics. b) (200)_c_ lattice plane of (1‐*x*)BNST‐xPT ceramics. c) the ratio of crystal parameters (*c/a*). d) domain morphology and SAED of P4bm symmetry in PT10. e) domain morphology and SAED of P4 mm in PT20. f) pinning effect in PT10. g) pining effect in PT20.

The XRD patterns of (1‐x)(75Na_0.5_Bi_0.5_TiO_3_‐25SrTiO_3_)‐xPbTiO_3_ ((1‐x)BNST‐xPT) (x = 5, 10, 20, 40 as PT5, PT10, PT20, PT40) ceramics are shown in **Figure** **1**a, and all the samples exhibit a pure perovskite structure. Figure 1b shows the magnified spectra in the range of 43.5–48.5° corresponding to {200}_pc_ diffractions, where subscript “pc” denotes pseudocubic coordinate system. In Figure 1b, the existence of tetragonal phase can be speculated from the splits of the (200) and (002) peaks, which is confirmed by the following Rietveld refinement. However, the peaks ≈46° are in different shapes, which may be attributed by other phases with different symmetries. Meanwhile, the crystal parameters are enlarged as the content of PT rising up because of large ion radius of Pb^2+^. The peak positions of (200) peaks for all the ceramics are almost unchanged, demonstrating a tiny change of *a/b* axis. However, (002) peak positions are obviously left shift, stemming from the elongation of *c* axis. The ratio of *c/a* is calculated and shown in Figure 1c, indicating *c/a* increasing with PT content.

In order to identify the phase structure and obtain more accurate crystal structure, Rietveld refinement has been carried out as shown in the Figure S1a‐d (Supporting Information), where the Bragg position has been marked in the figures by ticks. The coexistence of P4bm symmetry phase (tetragonal structure), P4 mm symmetry phase (tetragonal structure) and R3c phase (rhombohedral structure) in both PT5 and PT10 samples. With the PT content increasing, the R3c symmetry phase and P4bm symmetry gradually fade away and the P4 mm symmetry phase becomes dominate. With the content of PT increasing, both of PT20 and PT40 show the P4 mm single phase, and the results of refinement are shown in the Table S1 (Supporting Information). In general, R3c symmetry corresponds to the ferroelectric phase, and P4bm denotes to the relaxor exhibition in BNT‐based ceramics,^[^ ^22^, ^23^, ^24^ ^]^ while the P4 mm symmetry is a typical long‐range order ferroelectric phase commonly found in PT ceramics.

The morphology of crystal grains after thermally etching is shown in Figure S2a‐h (Supporting Information) and the EDS of PT20 indicates that the uniform composition again (Figure S2i‐n, Supporting Information). The domain structure is shown in Figure 1d,e. The coexistence of nano domains and long‐range order macro‐domains in PT10 was proved by TEM as Figure 1d. The lamellar structure was observed in PT10 and disordered regions are observed in the same grain. The selected area electron diffraction (SAED) patterns further proved the existence of P4bm symmetry denoted the relaxor ferroelectric phase. The P4bm symmetry is featured by the superlattice of 1/2(*ooe*) due to the *a* ^0^ *a* ^0^ *c* ^+^ oxygen octahedron tilting,^[^ ^25^, ^26^, ^27^, ^28^ ^]^ where the *o* means odd and *e* means even Miller index. The insert in Figure 1d is the SAED of PT10 along the direction of [001]_pc_ and the superlattice of 1/2(*ooe*) is marked in the pattern though the lamellar structure is dominate in the grain. In contrast, the domains in the PT20 also exhibit a lamellar structure, but the SAED pattern proves the P4 mm structure which is lack of superlattice due to the. According to previous report, the direct contact among the domains, which is called “domain intersection”, has been found in tetragonal phase ceramics and the domain switching is suppressed.^[^ ^29^ ^]^ The domain intersection effect exits in both PT10 and PT20 ceramics as shown in Figure 1f,g. In Figure 1f, some domains attempt to run across other domains, which means the domain pining effect appear and the pining region is sparse. However, the domain interaction is more complex in PT20, shown in Figure 1g. The domains are much smaller than those in Figure 1e and intense interaction occurs among each other. The intense domain pinning effect is inferred as one of the origins of high coercive field.^[^ ^30^ ^]^ And the supposition will be verified in ferroelectric measurements.

**Figure 2:**
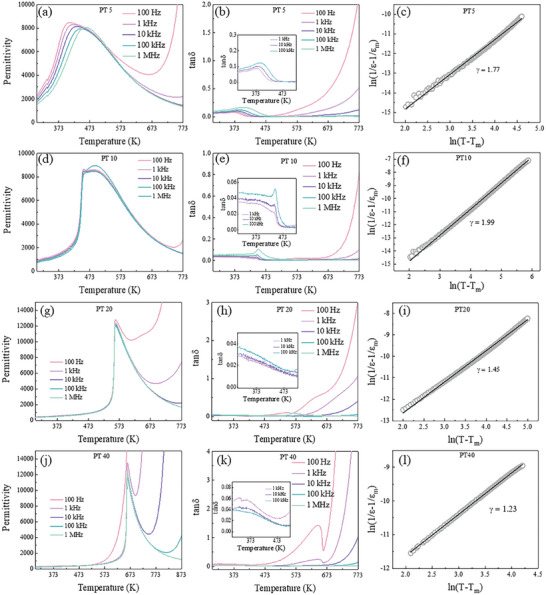
Temperature‐dependent permittivity, dielectric loss and Curie‐Weiss plots at 10 kHz of a‐c) PT5 d‐f) PT10 g‐i) PT20 j‐l) PT40.

The temperature dependence of permittivity and dielectric loss for the (1‐*x*)BNST‐*x*PT ceramics are shown in **Figure** **2**. All of the ceramics show the broad dielectric peaks and the *T_m_* (peak temperature in permittivity – frequency curve) gradually increase as the content of PT rises up. For PT5, the feature is that the *T_m_* increases with the frequency increases as shown in Figure 2a. The obvious frequency dispersion demonstrates that PT5 belongs to the relaxor ferroelectrics.^[^ ^31^, ^32^, ^33^ ^]^ The dielectric constant above *T_m_* obeys modified Curie‐Weiss law,^[^ ^34^ ^]^ as Equation 1 and the calculation is shown in Figure 2c.

(1) $$\frac{1}{\varepsilon }-\frac{1}{\varepsilon_{m}}=\frac{{\left(T-T_{m}\right)}^{\gamma }}{C}$$

where, ε is relative permittivity; ε_*m*_ is the maximum of relative permittivity; *T_c_* indicates the Curie temperature; *C* is the Curie constant and γ represents the degree of diffused phase transition. Generally, *γ* = 2 is corresponding to the complete disorder system. PT5 exhibits γ = 1.77 under 10 kHz, which implies relaxor behavior. Likewise, the dielectric loss spectrum of PT5 was typical by a diffused abnormal peak ≈375 K, corresponding to the permittivity plots (Figure 2b).

For PT10, temperature dependence of permittivity behaves are quite different from that of PT5, as shown in Figure 2d‐f. There are two peaks in the permittivity ≈ temperature curves for PT10, and the sharp peak appears at lower temperature and dispersive peak at higher temperature. According to the previous reports,^[^ ^35^, ^36^, ^37^ ^]^ such dielectric behavior repents the phase transition from normal ferroelectric P4 mm to relaxor ferroelectric P4bm with a diffused phase transition (DPT). *T*_C_ corresponding to the sharp peak and *T_m_* corresponding to dispersive peak are almost unchanged under the frequencies of 100 Hz, 1 kHz, 10 kHz and 100 kHz. The Curie‐Weise plots is shown in Figure 2f and the degree of relaxor is calculated as *γ* = 1.99. The dielectric loss of PT10 was slightly different from PT5, a sharp abnormal peak appears ≈455 K, which is corresponding to the depolarization.

The permittivity versus temperature spectrograms of PT20 and PT40 are semblable while different from PT5 or PT10. Sharp anomalous permittivity peaks appear at ≈573 and 673K, respectively, as shown in Figure 2g,j. The sharp peaks generally stem from macroscope domain and corresponds to the phase transition from ferroelectric to paraelectric.^[^ ^38^ ^]^ According to the Rietveld refinement, P4 mm ferroelectric phase is responsible for such temperature‐dependent permittivity behavior. The temperature‐dependent dielectric loss of PT20 and PT40 is shown in Figure 2h,k, respectively. Similar to PT10, the *T_m_* of PT20 and PT40 are almost frequency‐independent. However, Curie‐Weise plots of PT20 and PT40 show the γ is 1.45 and 1.23, as shown in Figure 2i,l, higher than complete ferroelectric‐paraelectric transition which is corresponding to the *γ* = 1. The slight diffuse behave may be account of the BNT in the solution. Bi^3+^ and Na^+^ in the A‐site break the long‐range order of macroscope ferroelectric domains, but the macroscope ferroelectric domains have not become into nano‐scope domains completely, which results in *γ* value higher than 1.

The above results suggest that the phase transition behavior of (1‐*x*)BNST‐*x*PT by the addition of PT. The (1‐*x*)BNST‐*x*PT solutions change from typical relaxor ferroelectrics with obvious frequency dispersion to the normal ferroelectrics with a slight diffused phase transition (DPT) when the content of PT increases. In addition, the complex electric modulus plots of (1‐*x*)BNST‐*x*PT ceramics are shown in Supporting Information (Figures S3‐S6, Supporting Information) and the exhibition of exponential Debye‐like relaxor in (1*‐x*)BNST‐*x*PT ceramics is eliminated.

### 2.2 Field‐Induced Deformation

**Figure 3:**
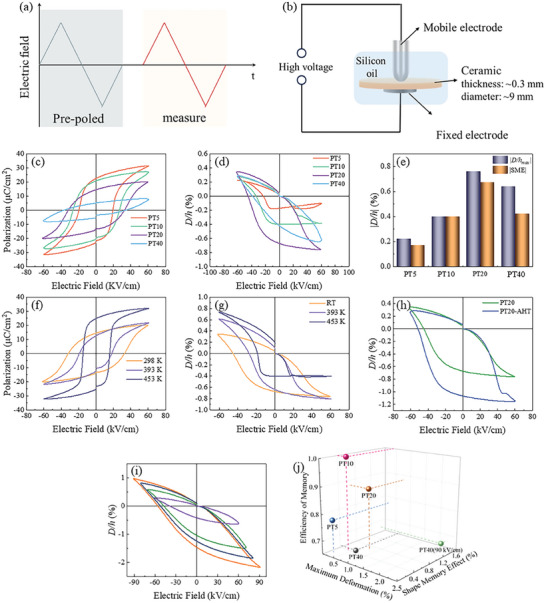
Ferroelectric properties of (1‐*x*)BNST‐*x*PT ceramics. a) the pre‐pole and triangle wave adopted in the measurements. b) Schematic diagram of electrostriction measurements. c) ferroelectric hysteresis loops of (1‐*x*)BNST‐xPT ceramics at 60 kV cm^−1^, 10 Hz. d) *D/h‐E* curves of (1‐*x*)BNST‐xPT ceramics at 60 kV cm^−1^, 10 Hz. e) absolute maximum of field‐induced deformation and field‐induced shape memory effect of (1‐*x*)BNST‐xPT ceramics. f) temperature‐dependent ferroelectric hysteresis loops of PT20. g) temperature‐dependent *D/h‐E* curves of PT20. h) enlarged field‐induced deformation and strain memory effect of PT20 after the high‐temperature ferroelectric measurement. i) *D/h‐E* curves in different electric field of PT40. (j) EOM of BNST‐xPT ceramics.

The ferroelectric property and field‐induced *D/h‐E* curves were measured at 10 Hz and are shown in the **Figure** **3**. The ferroelectric properties were measured and recorded after a switching pre‐poled as shown in the Figure 3a,b. The *P*‐*E* loops at of (1*‐x*)BNST‐*x*PT ceramics are shown in the Figure 3c. With the content of PT rising up, the coercive field (*E_c_*) increased. The ferroelectric hysteresis loops for PT5 and PT10 are both rectangular and saturated at 60 kV cm^−1^, while the loops with leaf‐like shapes for PT20 and PT40 ceramics are incompletely saturated. The *D/h‐E* curves are shown in the Figure 3d. It is noted that the *D/h‐E* curves are in asymmetrical shapes and negative *D/h‐E* appears when the electric field is positive. The maximum value of deformation increases when the content of PT rise up from 5% to 20% and then declines as the content of PT furtherly increases to 40%. The *D/h‐E* curves show a large field‐induced shape memory effect which means the remanent defomation when the external field is removed. The field‐induced shape memory effect also achieves the maximum at PT20. The PT20 exhibits the highest field‐induced deformation as 0.76% and the field‐induced shape memory effect as 0.67% at 60 kV as shown in Figure 3e For PT20, when temperature rising up, the domains are activated and switch more easily. The *E_c_* of PT20 is 32.7 kV cm^−1^ at room temperature and decreases to 15.6 kV cm^−1^ at 453 K. In addition, the ferroelectric hysteresis loops became saturated with a maximum polarization (*P_m_*) reached 32.0 µC cm^−2^, as shown in Figure 3f. Interestingly, the higher degree of polarization of PT20 at 453 K does not associate with a larger shape deformation and field‐induced shape memory effect. The maximum absolute deformation and field‐induced shape memory of PT20 drops to 0.4% at the same time, as shown in Figure 3g. What's more, an enhanced field induced deformation property appears in the sample which experienced high temperature tests (PT20‐AHT) as shown in Figure 3h. The *D/h‐E* curves of PT20‐AHT demonstrate a deformation of 1.14% which is 0.39% higher than the initial sample. The strain shape memory of PT20‐AHT is 1.07%, 0.40% higher than initial PT20. In addition, PT40 is enable to tolerate larger electric field and electromechanical response of the PT40 ceramic at higher external electric field increases rapidly, as shown in Figure 3i. When the external electric field rising up to 90 kV cm^−1^, the maximum deformation is 2.17% and field‐induced shape memory effect is 1.46%. The efficiency of memory (*EOM*) is defined by Equation (2) as following:

(2) $$EOM=SME/Deformation_{max}$$

where the *SME* is field‐induced shape memory effect and *Deformation_max_* is maximum *D/h*. Figure 3j shows the *EOM*, *SME* and *Deformation_max_* of BNST‐*x*PT ceramics. It is noticed that a large *D/h* or field‐induced shape memory effect does not mean high efficiency. The PT10 ceramic shows an efficiency of 100% while the field‐induced deformation is smaller than PT20 and PT40 at 60 kV cm^−1^, 10Hz. The field‐induced deformation properties of PT20 is largest among the (1‐*x*)BNST‐*x*PT ceramics at the same measurement condition and the efficiency of the PT20 is 88.2%. Whether PT40 ceramic is measured at 60 kV cm^−1^ or 90 kV cm^−1^, both are less than 70% efficient.

### 2.3 Discussion

**Figure 4:**
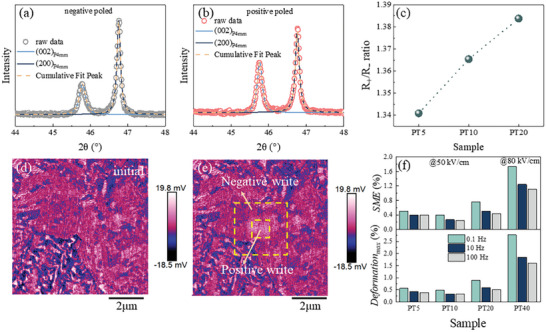
Different response of PT20 to negative electrode and positive electrode and frequency‐dependent electromechanical response. a) (002)c/(200)c of negative poled surface. b) (002)c/(200)c of positive poled surface. c) R+/R‐ ratio of PT5, PT10 and PT20 at 40 kV cm^−1^. d) PFM of unpoled PT20. e) write and read test of PT20 by PFM, where the bigger box shows the negative write and smaller box shows the positive write. f) frequency‐dependent field‐induced deformation properties of (1‐*x*)BNST‐*x*PT ceramics.

To interpret the negative *D/h* and the large strain shape memory effect, the crystal structure of poled ceramics was characterized by XRD. An obvious difference between the two surfaces of the poled samples. The fine scan of PT20 between 44° and 48° [(002) and (200) peaks] are shown in **Figure** **4**a,b, where the sample experienced an electrical triangular wave with the amplitude of 40 kV cm^−1^. When the sample undergoes a ferroelectric measurement, the surface which was adjacent the positive electrode is marked as positive‐poled surface and the opposite side is marked as negative‐poled surface. To describe the polarization degrees of the negative‐poled and positive‐poled surface, we define a polarization parameter *R* (*R*_+_ for positive‐poled surface, *R*_‐_ for negative‐poled surface) as *R*  = *I*_002_/*I*_200_  where, *I*_002_and *I*_200_ are the intensities of (002) and (200) diffractions for poled surface. And $R_{0}=I_{002}^{0}/I_{200}^{0}$ is for unpoled powders, where $I_{002}^{0}$ and $I_{200}^{0}$ are the intensities of (002) and (200) diffractions for unpoled powders. It is clear that $I_{002}^{0}/I_{200}^{0}\approx 0.5$ for unpoled powders.

Negative‐poled surface of PT20 exhibits a very weak polarization as the *R*_‐_ value is 0.49, which is almost the same with that of unpoled P4 mm symmetry feature. At the same time, the *R_+_* value is 0.68, which means that the positive‐poled surface is poled more intensively than negative‐poled surface. The polarization asymmetry between the two surfaces can be characterized by the ratio of *R*_+_/*R*_‐_. As shown in Figure 4c and *R*_+_/*R_‐_* ratio increases with PT content increasing, and *R*_+_/*R_‐_* value reach 1.38 for PT20 sample. The calculation is ploted in Figure 4c.

The XRD patterns of poled PT5, PT10 and PT40 are shown in Figure S7a‐f (Supporting Information). Obvious peak shape variation is shown in the XRD pattern of PT5 and PT10, respectively. Combining with the phase structure characterization as previously mentioned, the PT5 and PT10 ceramics experiment a phase transition P4bm to P4 mm and the switching from *a* domains to *c* domains occurs in all the samples. The polarization difference between the two surfaces was also reported by He Xiang et al.^[^ ^18^ ^]^

To further verify the response between different polarization degree to positive and negative stimulation, electric field writing and reading test by out‐of‐plane piezo response are shown in Figure 4d,e. First, an 8 µm × 8 µm square was recorded (Figure 4d). The blocks of varying shades of color demonstrates a random distribution of domains. By applying a −10 V stimulation, a negative square (5 µm × 5 µm, outside box in Figure 4e) was written on the surface of polished ceramics and then +10 V stimulation was applied in a smaller square (3 µm × 3 µm, inside box in Figure 4e). Through comparing the written area and initial surface, the negative writing area is almost unchanged while the positive writing area exhibits an obvious domain switching. The PFM of PT5 and PT10 are shown in Figure S7g,h (Supporting Information). The negative writing area was obvious in PT5 and slightly weak in PT10. All the samples were sensitive to positive simulation. The response of domain switching to positive and negative DC with the same amplitude agreed with XRD patterns. The different degree of polarization in two surfaces is the basis of bending deformation. Similar difference also can be observed by SEM as shown in Figure S8 (Supporting Information).

In addition, the electromechanical response was dependent with trigger wave frequency (Figure 4f). A huge enhancement is accomplished when the trigger wave frequency is decreased from 100 Hz to 0.1 Hz, where PT20 ceramics shows a maximum deformation of −0.88% and the shape memory effect of 0.76% at 50 kV cm^−1^ and the PT40 shows a maximum deformation of 2.77% and the shape memory effect of 1.16% at 80 kV cm^−1^. The frequency‐dependent *D/h‐E* curves are shown in Figure S9 (Supporting Information).

According to the above phenomenon, the different responses to the negative and positive electrodes result in the bending deformation of (1‐*x*)BNST‐xPT ceramics under an external electric field. After the pre‐poling, the surfaces of ceramic exhibit different polarization degree and bending deformation appears. Then the external field change direction, the convex ceramic gradually reverts to plate and finally translates to concave shape. For the top indenter, the switching from convex to concave is a negative displacement which results in a negative s *D/h‐E* curves. When the electric field drops to zero, the ceramics still maintains a concave state, that is the negative field‐induced shape memory effect.

**Figure 5:**
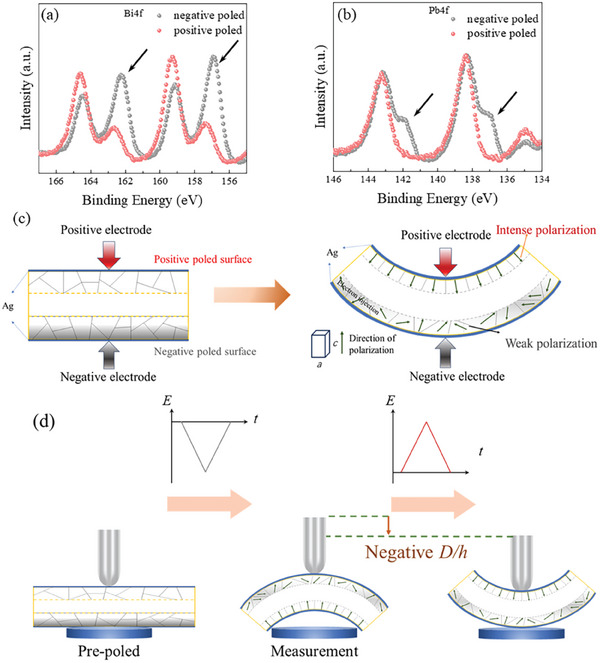
Mechanism of bending deformation over (1‐*x*)BNST‐xPT ceramics. XPS fine spectra of a)Bi4f b) Pb4f on negative and positive poled surface c) schematic diagram of bending deformation mechanism. d) schematic diagram of negative *D‐h* and memory effect.

To elucidate the effect of positive and negative electrodes on the asymmetry polarization behavior, the chemical states of both surfaces were characterized by X‐ray photoelectron spectroscopy (XPS). The fine spectra of Bi and Pb for PT20 are shown in **Figure** **5**a,b. For Bi4f fine spectra, two doublets of 4f_7/2_ and 4f_5/2_ are responding to the oxide state (≈159.3 eV and ≈164.6 eV) and metallic state (≈156.9 eV and ≈162.3 eV) [arrowed in Figure 5a].^[^ ^39^, ^40^ ^]^ The negative‐poled surface exhibits enhanced metallic state. The intense reduction also appears in Pb4f spectra. In Pb4f spectra, the peaks at ≈143.2 eV (4f_7/2_) and ≈138.4 eV (4f_5/2_) demonstrate the oxide‐state Pb and the ≈142 eV and ≈137.15 eV belonged to the metallic state [arrowed in Figure 5b]. By contrast, in positive‐poled surface, Bi shows a weaker reduction, and there was no indication that the Pb had been reduced (Figure 5a,b).^[^ ^41^, ^42^ ^]^ However, there was little difference between the two surfaces in Sr3d, Ti 2p and Na1s spectra (Figure S10, Supporting Information). Similar difference in PT5, PT10 and PT40 are shown in Figures S11–S13 (Supporting Information).

It could be speculated that the reduction of Bi and Pb stem from the charge injection from negative electrode. When an external electric field is applied, the dipoles orientate along the field and generate a certain intensity of polarization to cancel out the external electric field. In general, the polarization degree is positively correlated with the electric field. When the chemical reduction is taken into account, the electric field assigned to drive the domain switching declines and results in a weak polarization. According to the previous reports, the metallic Bi would cause degradation of the ferroelectric properties.^[^ ^43^ ^]^ In addition, when the coercive field is large enough, the difference between two surfaces could be amplified because the negative poled surface is almost in unpoled state like PT20 and PT40. As for the PT5 and PT10, because of the lower coercive field, the chemical reduction resulted limited impact and both surfaces of PT5 and PT10 were intensely poled. Further notice, when the different polarization appears, the bending deformation is related to the mismatch between *a* axis and *c* axis, larger *c/a* means larger bending deformation (illustrated in Figure 5c). Based on above results, when the content of PT rose up, the difference between two surfaces goes more obvious and *c/a* is larger which resulted in a larger bending deformation.

On this basis, the large field‐induced shape memory effect can be further demonstrated [Figure 5d]. It has been illustrated that the (1‐*x*)BNST‐*x*PT ceramics are able to bend in the positive electrode direction. The samples turn into convex shape because that the bottom electrode work as the positive electrode during pre‐poling. When the ferroelectric measurements start, the positive electrode is the top indenter thus the ceramics become into concave shape. Although the external electric field has decreased to zero, the concave shape is maintained which is the birth of field‐induced shape memory effect. The stable concave at zero has been proved by asymmetric polarization (Figure 4).

## 3 Conclusion

In summary, the (1‐*x*)BNST‐xPT ferroelectric ceramics fabricated by solid state method show pure perovskite structure. The electromechanics response of (1‐*x*)BNST‐xPT ceramics are specific with hysteresis‐like *D/h‐E* curves. The asymmetry curves stem from the bending deformation and show giant field‐induced deformation and strain shape memory effect. It is demonstrated that the bending deformation is original from the restrained polarization in negative poled surface which is inferred by chemical reduction state in negative poled surface. In addition, as the content of PT rise up, the difference between two surfaces is enhanced and the crystal structure shows a larger *c/a*, which makes the shape deformation and shape memory effect larger. The normal deformation achieves 2.77% and field‐induced shape memory effect is 1.75% at 80kV cm^−1^ and 0.1 Hz in the ceramic with the content of PbTiO_3_ is 40%. It is indicated that the (1‐*x*)BNST‐*x*PT ceramics is a kind of potential electrochemical actuator candidate.

## 4 Experimental Section

(1‐*x*)(75Na_0.5_Bi_0.5_TiO_3_‐25SrTiO_3_)‐xPbTiO_3_ ((1‐*x*)BNST‐xPT) (x = 5, 10, 20, 40 as PT5, PT10, PT20, PT40) ceramics were prepared by conventional solid state method. Raw reagent grade powders Na_2_CO_3_, Bi_2_O_3_, SrCO_3_, TiO_2_ and PbO were weighted in the light of the stoichiometric ratio and then ball milled with zirconia ball and ethanol media in a zirconia jar at 300 rpm for 12 h. The mixed powders were dried at 80 °C and then calcined at 900 °C for 4 h. The calcined powders were ball milled for 12 h again and dried at 80 °C. the powders were mixed with 5 wt.% PVA as binder and pressed into a green body of 10 mm in diameter at uniaxial pressure. The samples were calcined at 600 °C for 1 h to remove the PVA and then sintered in closed alumina crucibles at 1150 °C for 3 h.

The crystal structures of the samples were characterized by X‐ray diffractometer (XRD, Philips X‐Pert Pro Diffractometer, The Netherlands). The domain morphology was observed by transmission electron microscope (TEM, Talos F200X G2 TEM; Thermo Fisher Scientific, America). The response to negative write and negative write in the surfaces of ceramics was carried by the piezo force microscope (PFM, Dimension Fastscan; Bruker, German). To measure the electrical properties, the samples were polished and painted with silver paste on the both sides. The dielectric properties were measured at atmosphere in a temperature range of room temperature (RT)‐600 °C by an impedance analyzer (Agilent 4294A, The USA) coupled with a temperature‐controlled system. The ferroelectric hysteresis loops and *D/h‐E* curves were carried out using the ferroelectric analysis system (TF‐analyzer 3000, aixACCT, German) applied a temperature‐controlled system and SOIS laser module. The field‐induced deformation was calculated by *Deformation*  = *D*/*h* , where *D* is the displacement of top indenter and *h* is the thickness of the sample. The element and electron motivation were studied by XPS (Esca Xi+, Thermo Fisher Scientific, America)

## Conflict of Interest

The authors declare no conflict of interest.

## Author Contributions

M.L. performed conceptualization (lead), data curation (lead), formal analysis (lead), wrote the original draft (lead). W.L. performed funding acquisition (lead), wrote, review and edited the final manuscript (equal). W.C. performed data curation (supporting). N.X. performed formal analysis (supporting). W.L. performed data curation (supporting). W.F. wrote, review and edited the final manuscript (equal).

## Supporting information



Supporting Information

## Data Availability

The data that support the findings of this study are available from the corresponding author upon reasonable request.
