# Supplementary material for: Large Electromechanical Response and Field‐Induced Shape Memory Effect in Ferroelectric Ceramics
Source: Adv Sci (Weinh). 2025 Jan 29;12(11):2410580. doi: 10.1002/advs.202410580 (PMC11923892; doi:10.1002/advs.202410580)
Supplement: Supplementary file 1 — Supporting Information [file ADVS-12-2410580-s001.docx]

Supporting Information

**Large electromechanical response and field-induced shape memory effect in ferroelectric ceramics**

Menglu Li, Weili Li*, Wenping Cao, Nuo Xu, Wenqi Li and Weidong Fei*


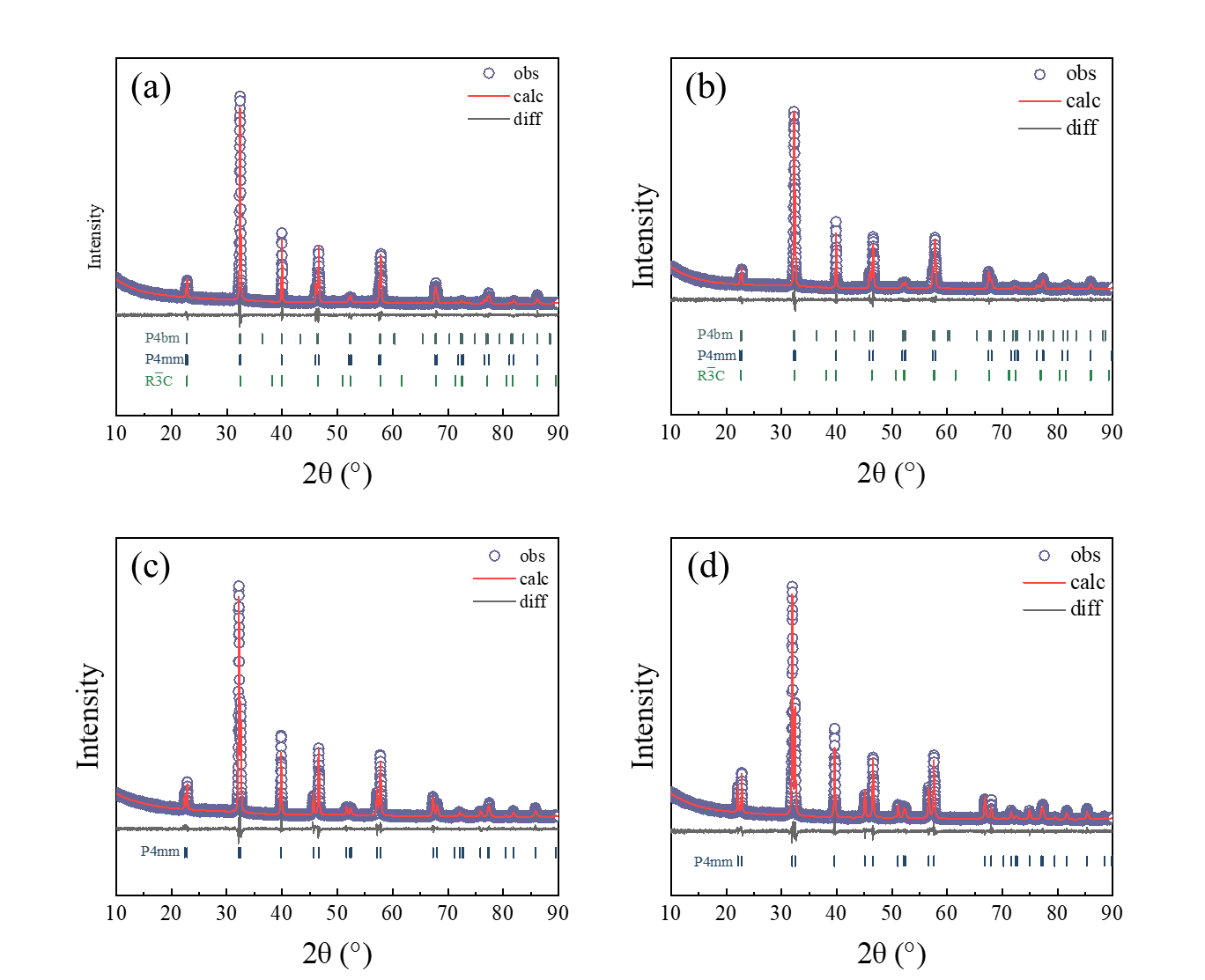


Figure S1. Rietveld refinement of (1-*x*)BNST-*x*PT ceramics (a) PT5 (b) PT10 (c) PT20 (d) PT40

Table S1. Refinement results of (1-*x*)BNST-*x*PT ceramics

|  | Symmetry | a (Å) | c (Å) | Content (%) | Rp (%) | GOF |
| --- | --- | --- | --- | --- | --- | --- |
| PT5 | P4bm | 5.51495 | 3.92697 | 34.67 | 5.824 | 1.71 |
|  | P4mm | 3.89295 | 3.93976 | 60.84 |  |  |
|  | R$\bar{3}$c | 5.52430 | 13.53308 | 4.49 |  |  |
| PT10 | P4bm | 5.51353 | 3.93573 | 31.13 | 5.639 | 1.35 |
|  | P4mm | 3.89202 | 3.95365 | 66.63 |  |  |
|  | R$\bar{3}$c | 5.53599 | 13.54198 | 2.23 |  |  |
| PT20 | P4mm | 3.89364 | 3.97174 | 100 | 5.930 | 1.81 |
| PT40 | P4mm | 3.89814 | 4.0179 | 100 | 6.617 | 1.59 |


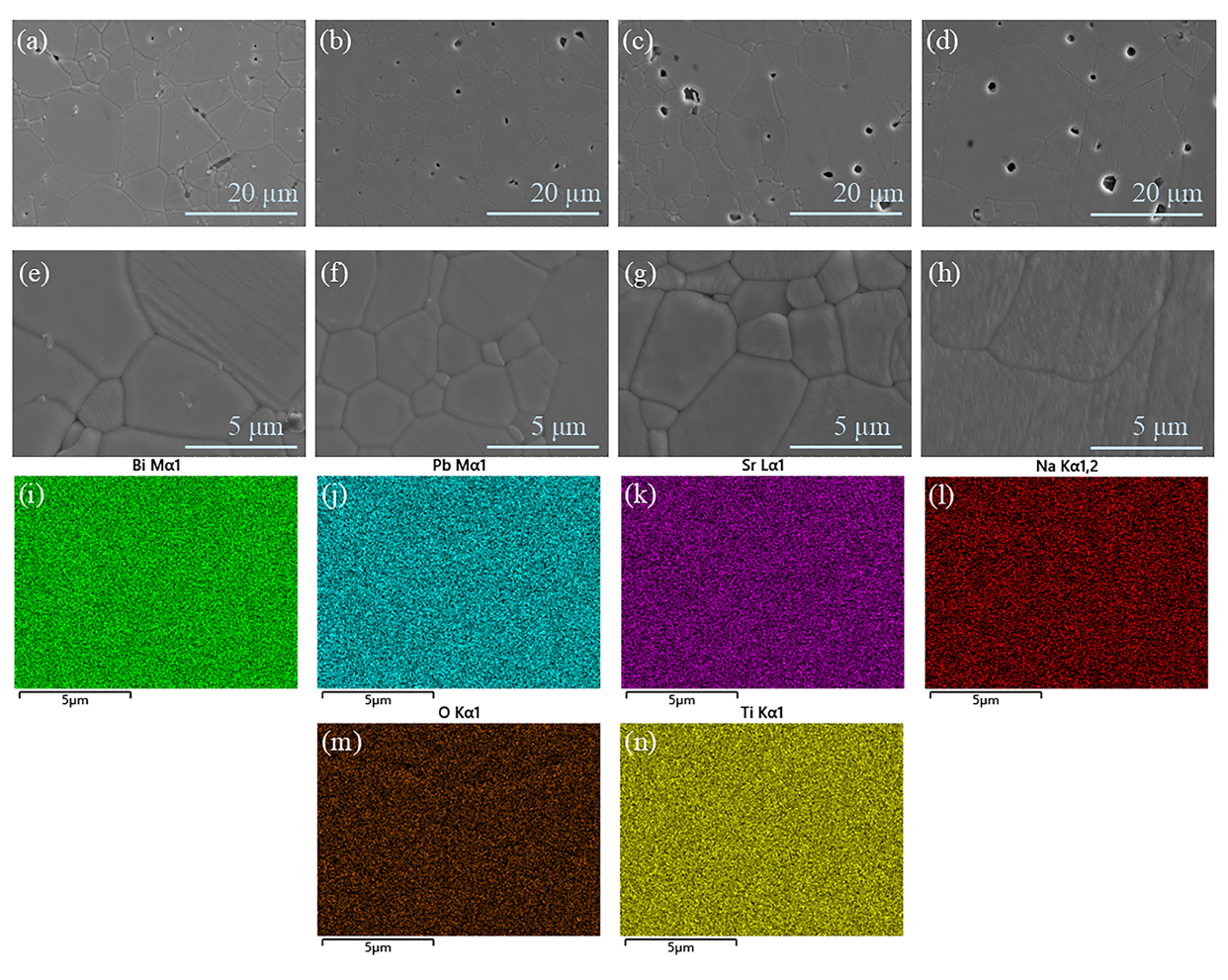


Figure S2 Morphology of (1-*x*)BNST-*x*PT ceramics. Grain morphology of (a) PT5 (b) PT10 (c) PT20 (d) PT40; high-magnification morphology of (e) PT5 (f) PT10 (g) PT20 (h) PT40; and EDS of PT20 (i) Bi

(j) Pb (k) Sr (l) Na (m) O (n) Ti


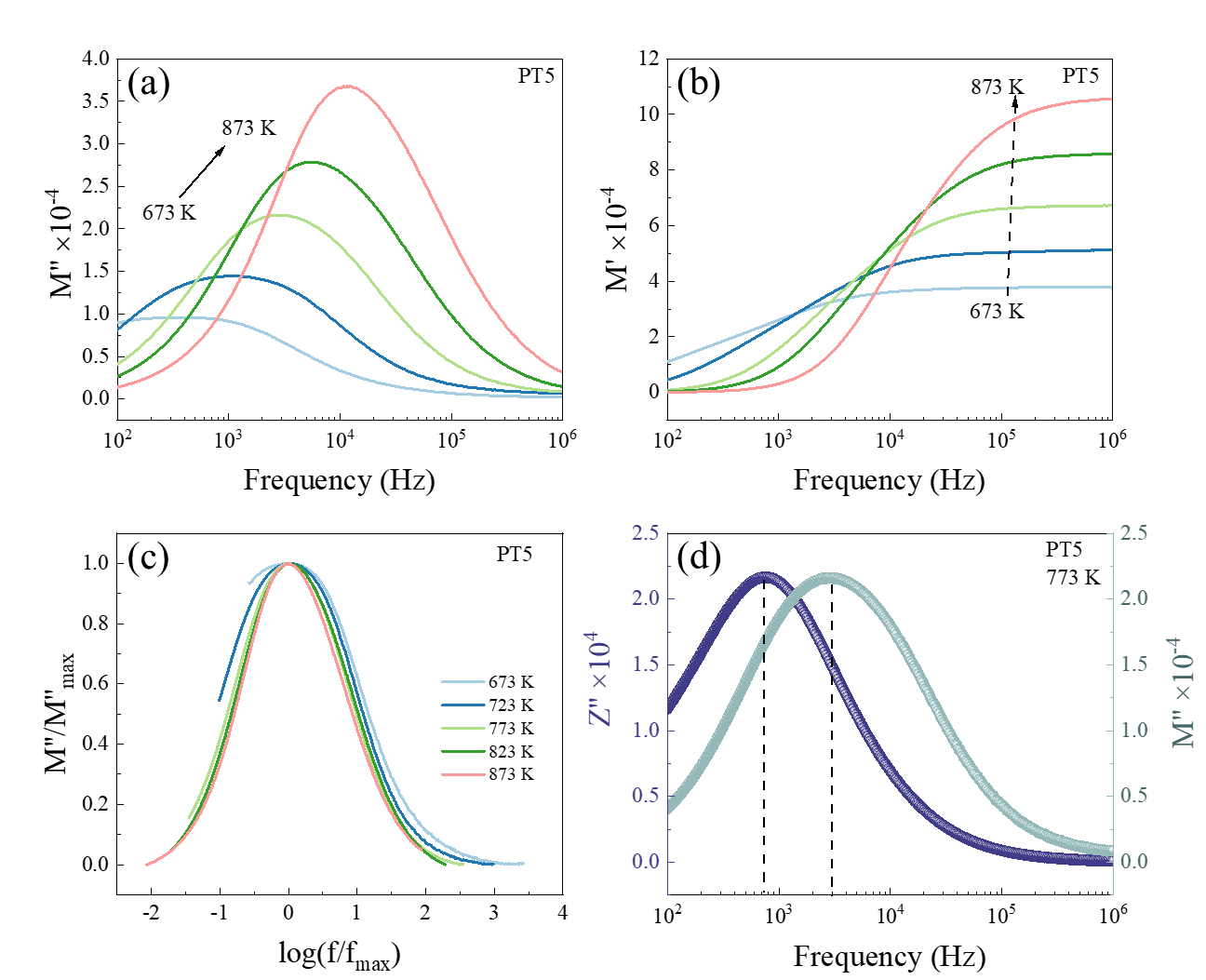


Figure S3 Complex electric modulus plots of PT5 ceramics (a) real part of electric modulus (b) imaginary part of electric modulus (c) imaginary of impendence and modulus versus frequency (d) normalized imaginary of electric modulus


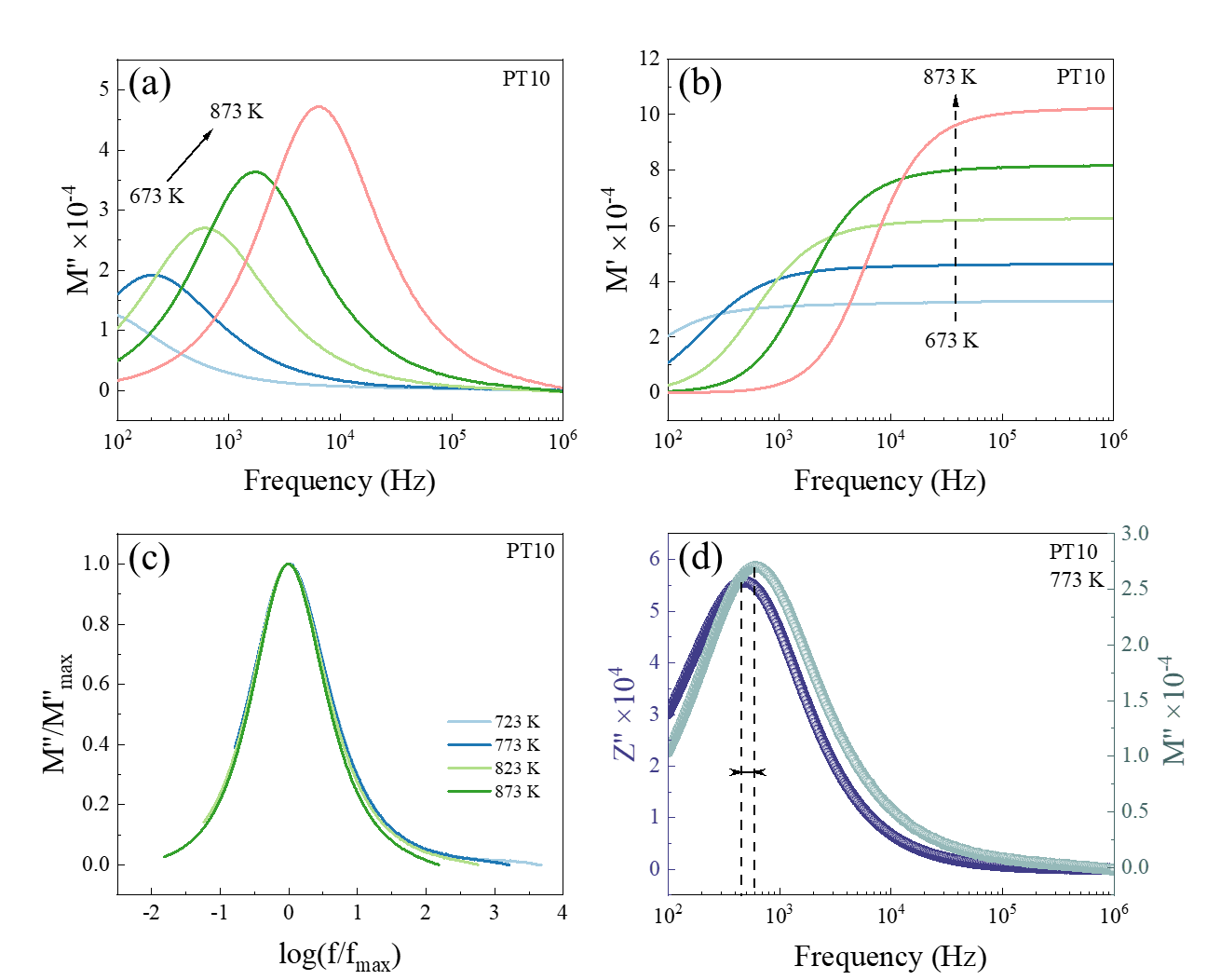


Figure S4 Complex electric modulus plots of PT10 ceramics (a) real part of electric modulus (b) imaginary part of electric modulus (c) imaginary of impendence and modulus versus frequency (d) normalized imaginary of electric modulus


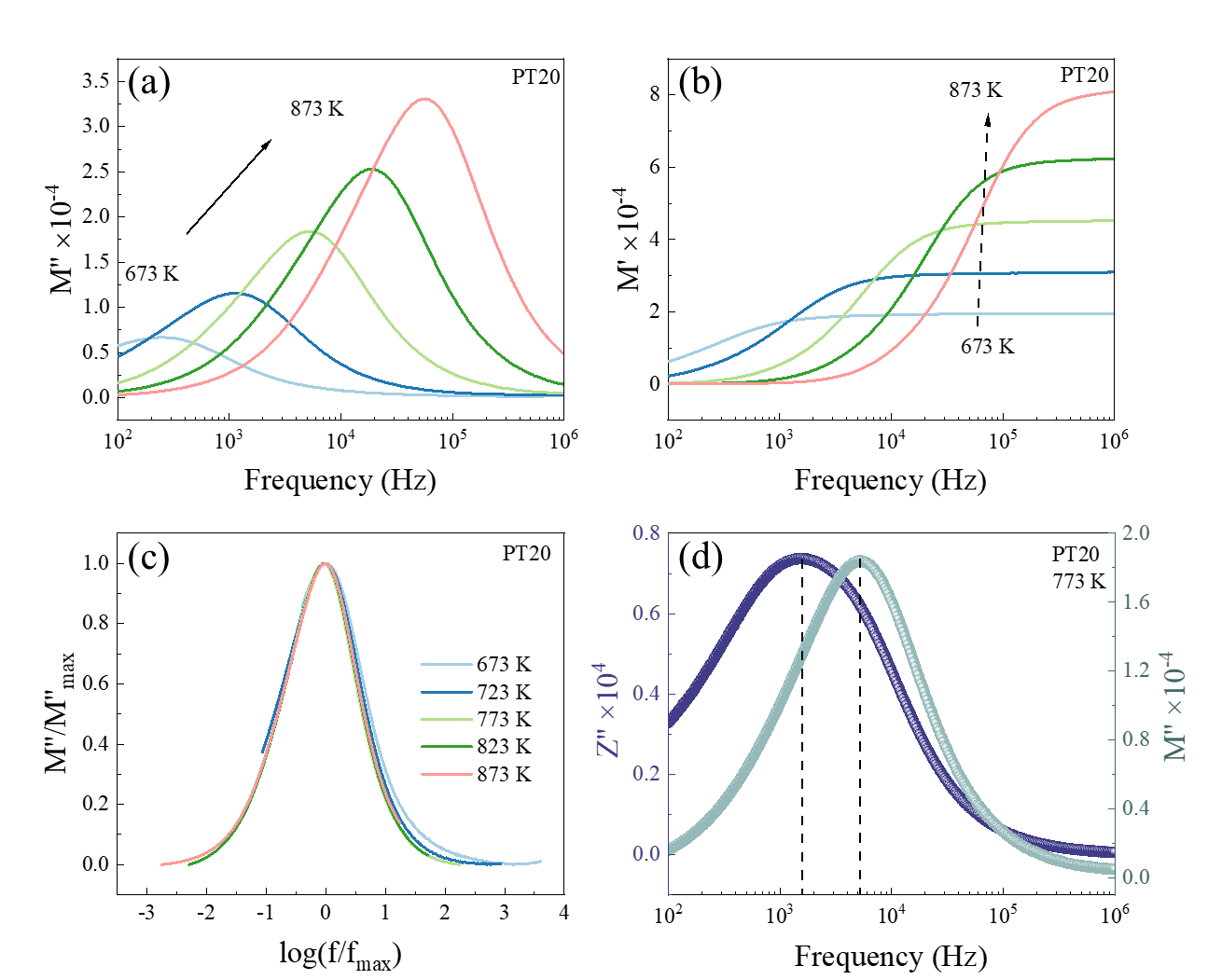


Figure S5 Complex electric modulus plots of PT20 ceramics (a) real part of electric modulus (b) imaginary part of electric modulus (c) imaginary of impendence and modulus versus frequency (d) normalized imaginary of electric modulus


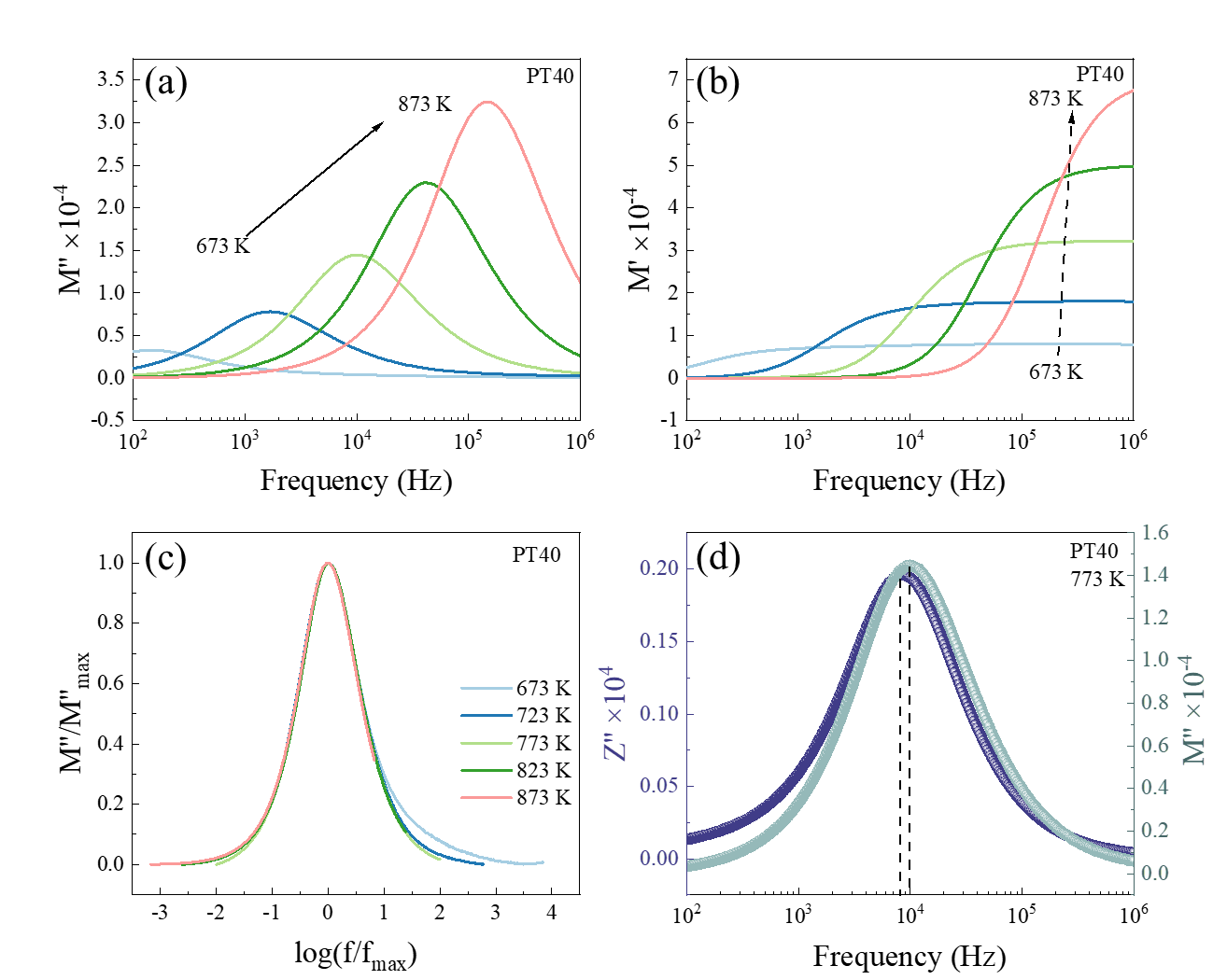


Figure S6 Complex electric modulus plots of PT40 ceramics (a) real part of electric modulus (b) imaginary part of electric modulus (c) imaginary of impendence and modulus versus frequency (d) normalized imaginary of electric modulus

All the samples process a sigmoidal step in the real part (M') of electric modulus and show a similar frequency dependence. It’s can be speculated that the polarization is negligible because that the minimum values of M' approach zero at low frequency.^[1]^

According to the Kohlrausch- Williams- Watts (KWW) function and imaginary part of electric modulus (M''), all the samples are not exponential Debye-like relaxor.^[2]^

The single peaks are overlapped perfectly in the normalized plots of imaginary electric modulus versus frequency, which denotes that the diffused behavior combines a temperature-independent dynamic process. ^[2, 3]^

As for the comparison of imaginary impedance(Z") and imaginary electric modulus(M") at 773 K, the peaks of Z" and M" are distinguish and proved that charge carriers process short-range motion in the diffuse mechanism.^[4]^


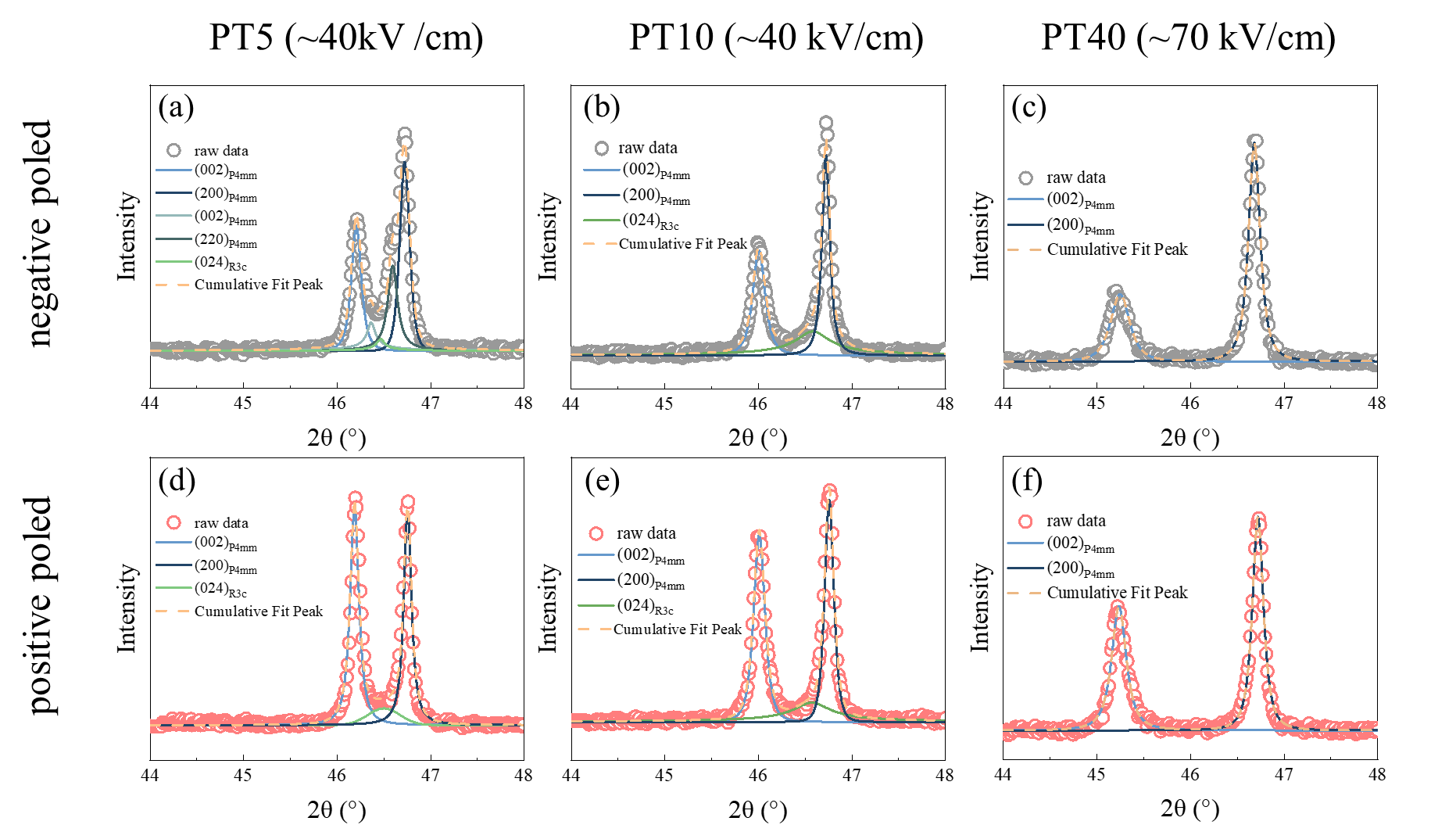


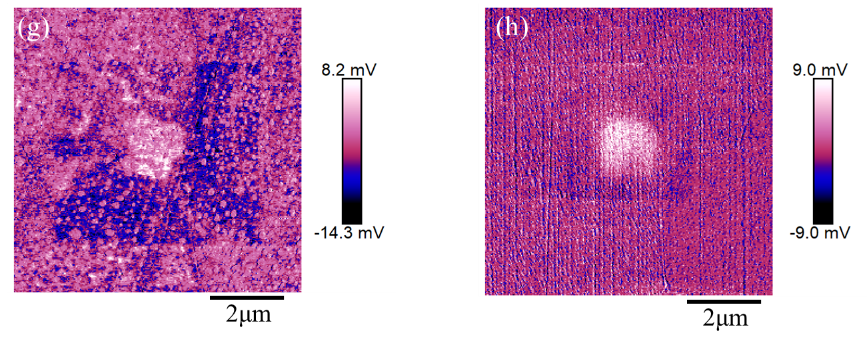


Figure S7 (002)_c_/(200)_c_ of negative poled surface (a) PT5 (~40 kV/cm)

(b) PT10 (~40 kV/cm) (c) PT40 (~70 kV/cm)

(002)_c_/(200)_c_ of positive poled surface (d) PT5 (~40 kV/cm)

(e) PT10 (~40 kV/cm) (f) PT40 (~70 kV/cm)

response of (g) PT5 (h) PT10 to negative electrode and positive electrode, where where the bigger box shows the negative write and smaller box shows the positive write


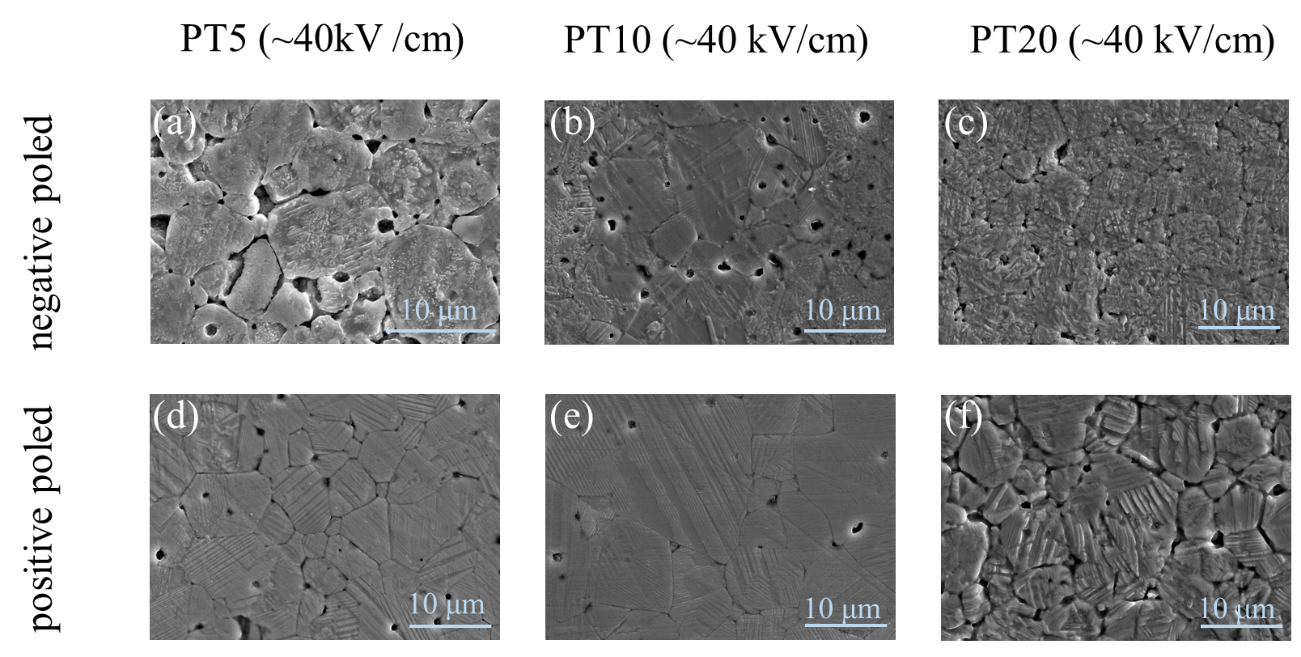


Figure S8 Domain morphology of negative poled (1-*x*)BNST-*x*PT ceramics (a) PT5 (b) PT10 (c) PT20 and domain morphology of positive poled (1-*x*)BNST-*x*PT ceramics (d) PT5 (e) PT10 (f) PT20


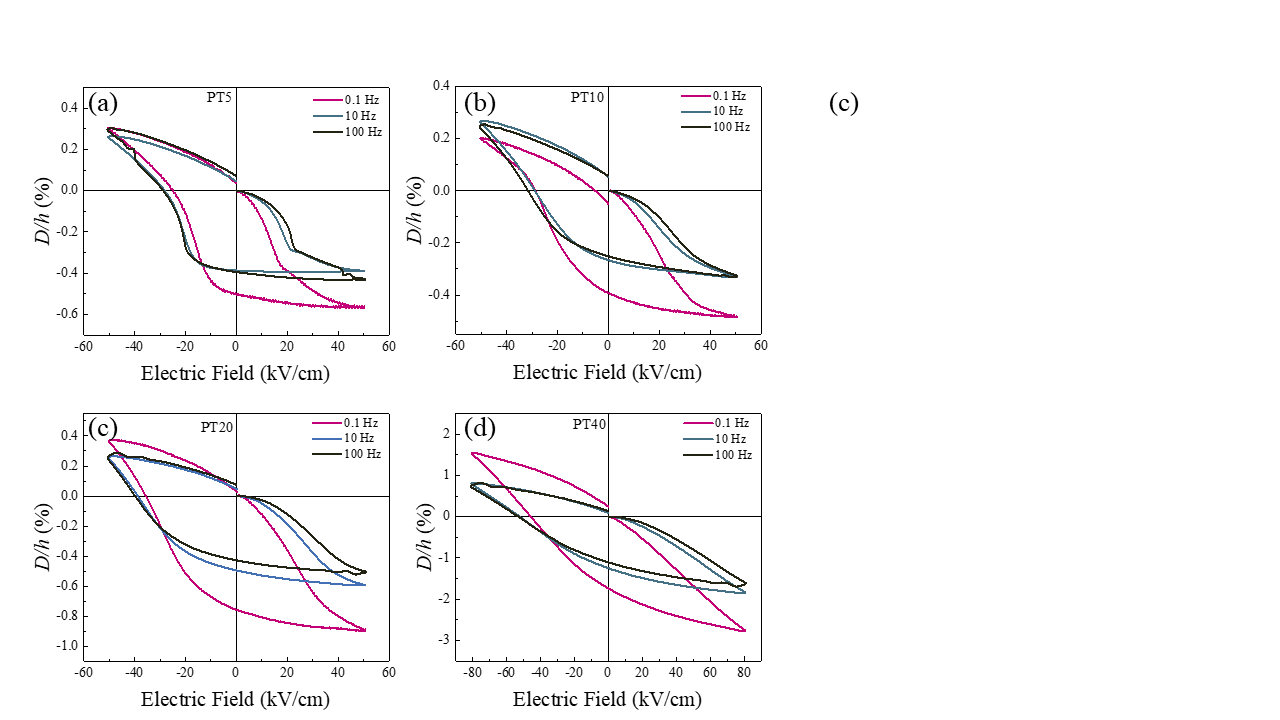


Figure S9 Frequency-dependent *D/h-E* curves of (a) PT5 (b) PT10 (c) PT20 (c) PT40


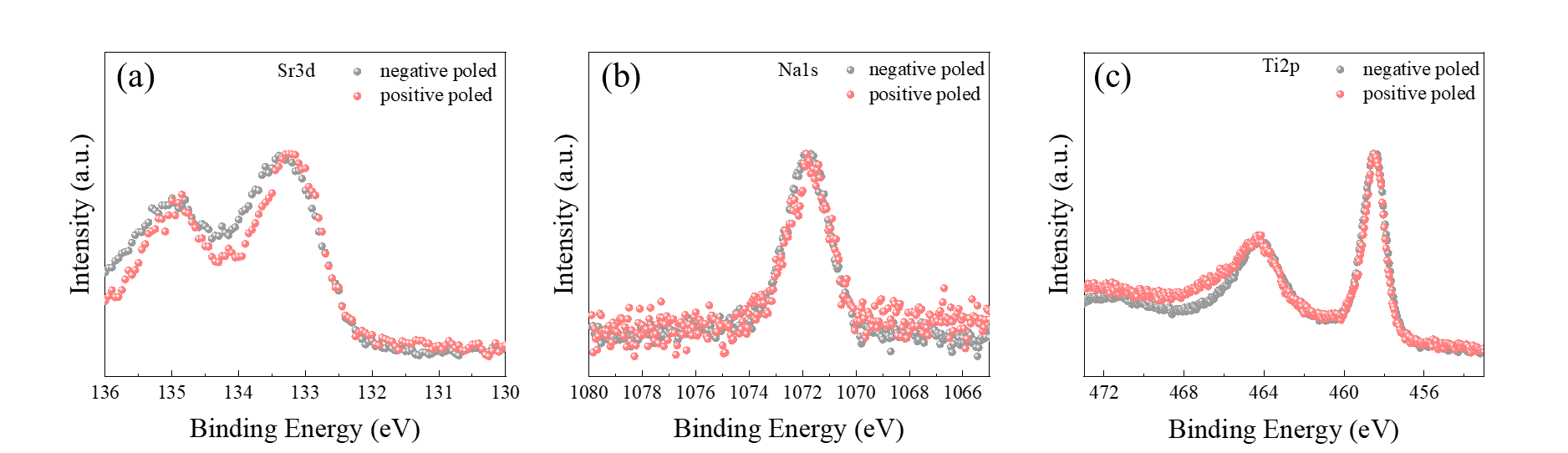


Figure S10 XPS fine spectra of PT20 (a) Na1s (b) Sr3d (c) Ti2p


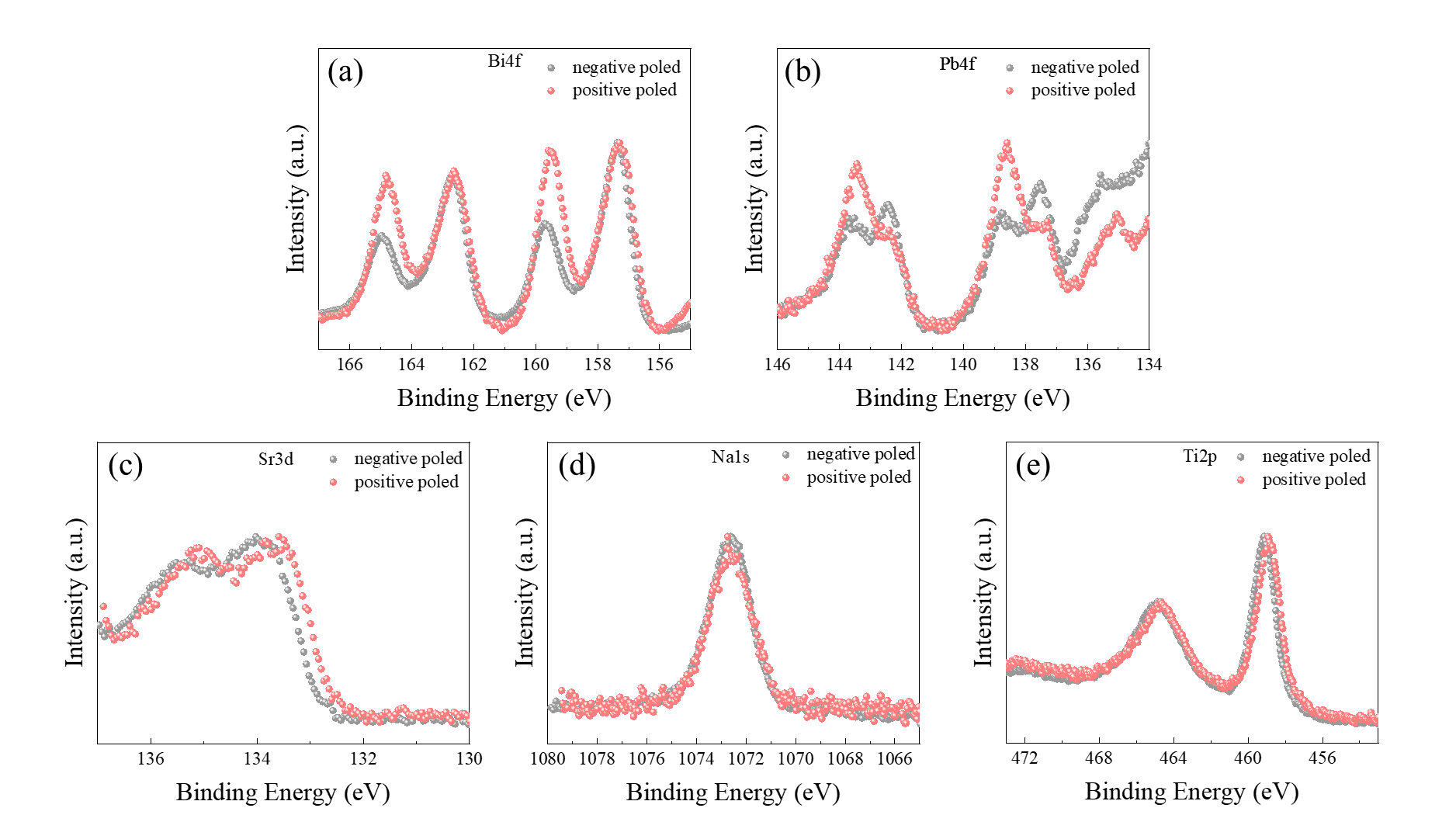


Figure S11 XPS fine spectra of PT5 (a) Bi4f (b) Pb4f (c) Sr3d (d) Na1s (e) Ti2p


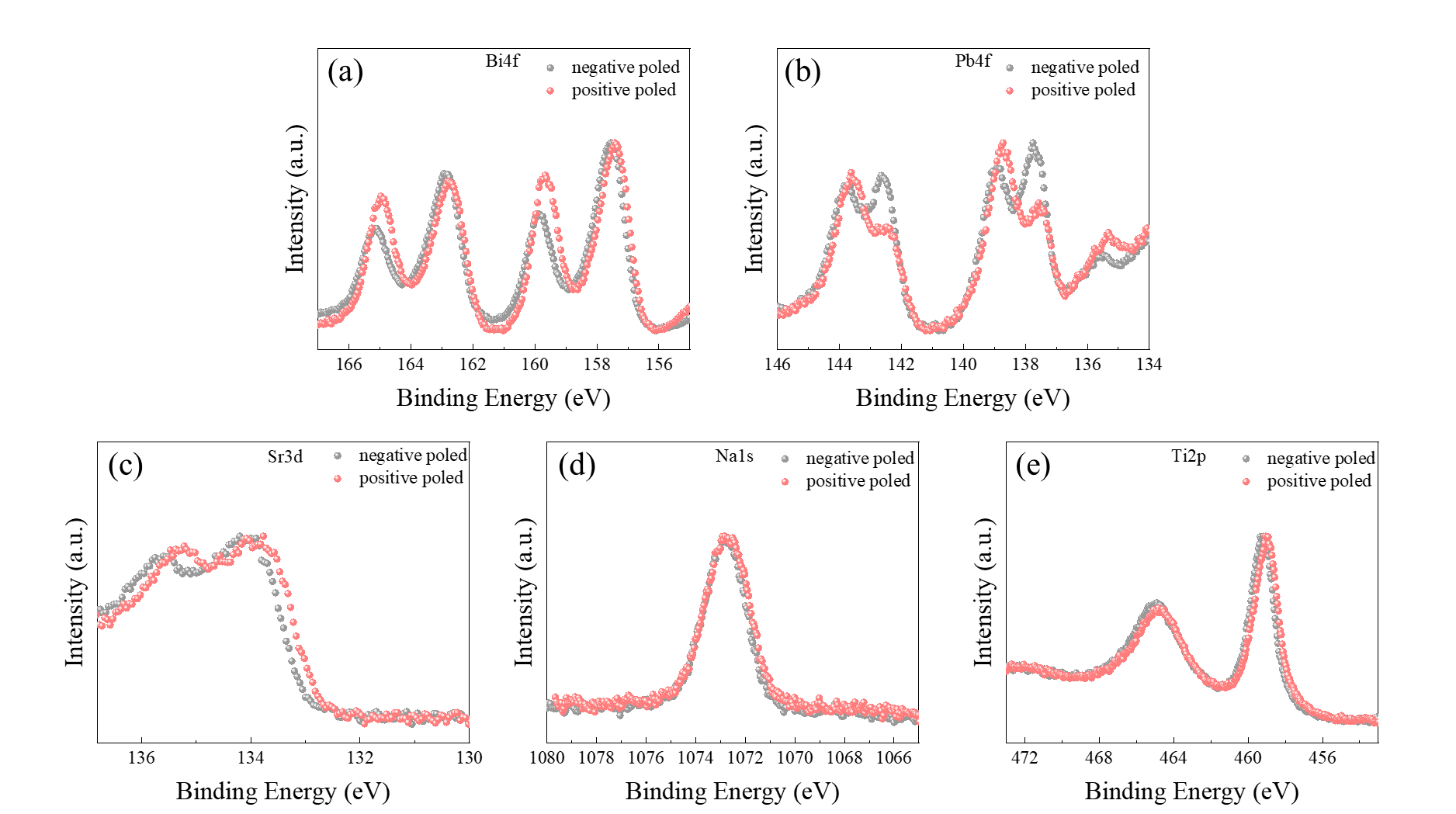


Figure S12 XPS fine spectra of PT10 (a) Bi4f (b) Pb4f (c) Sr3d (d) Na1s (e) Ti2p


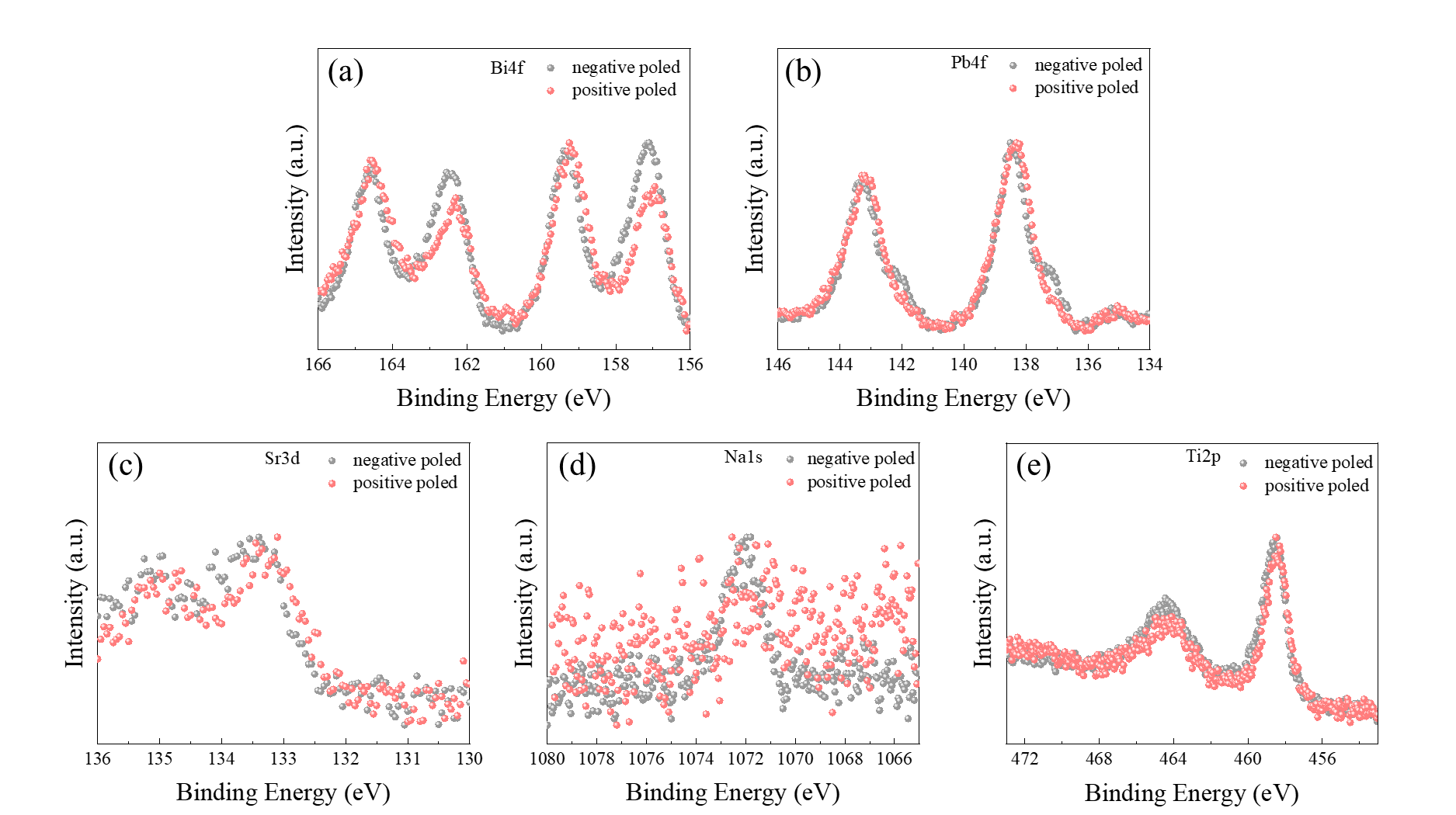


Figure S13 XPS fine spectra of PT40 (a) Bi4f (b) Pb4f (c) Sr3d (d) Na1s (e) Ti2p

**Reference**

[1] K. Yoshii, N. Ikeda, *J ALLOY COMPD* **2019**, *804*, 364.

[2] W. Kleemann, M. D. Glinchuk, V. Westphal, *PHYS REV LETT* **1992**, *68*, 847.

[3] Ashima, S. Sanghi, A. Agarwal, Reetu, N. Ahlawat, Monica, *J APPL PHYS* **2012**, *112*, 14110.

[4] F. Li, D. Lin, Z. Chen, Z. Cheng, J. Wang, C. Li, Z. Xu, Q. Huang, X. Liao, L. Chen, T. R. Shrout, S. Zhang, *NAT MATER* **2018**, *17*, 349.
